# Dataset: COVID-19 epidemic policy and events timeline (Sweden)

**DOI:** 10.1016/j.dib.2021.107698

**Published:** 2021-12-13

**Authors:** Tobias Olofsson, Andreas Vilhelmsson

**Affiliations:** a Department of Sociology, Lund University, Sweden; b Department of Laboratory Medicine, Division of Occupational and Environmental Medicine, Lund University, Sweden

**Keywords:** COVID-19, Sweden, Timeline, Qualitative data, Archival material, Swedish strategy, Pandemic governance, Public Health

## Abstract

The Swedish approach to managing the 2020-2021 COVID-19 pandemic has received significant attention in international scholarly work and press. For this dataset, we have reviewed governmental and media archives to build a detailed timeline that chronicles significant policies, interventions, and events in the Swedish management of COVID-19. The dataset contains summary descriptions of what took place, when it happened, and who the principal actors involved were. Links to primary sources are provided for each entry. Because of the level of detail and saturation, the dataset offers a detailed account of Swedish pandemic governance and will benefit anyone working on Swedish pandemic management or doing comparative work between Sweden and other jurisdictions.

## Specifications Table

**Table:** 

Subject	Social Science
Specific subject area	Timeline of government interventions and events regarding the COVID-19 pandemic in Sweden January 31, 2019, to June 3, 2021.
Type of data	Table
How data were acquired	Archival data from government and media archives
Data format	Raw Summarized
Parameters for data collection	Archival materials were collected, read, and summarized based on an assessment of whether the material described a policy or an event of relevance for the Swedish management of the COVID-19 pandemic.
Description of data collection	Systematic review of archival materials in publicly accessible online archives belonging to the government (regeringen.se) and Swedish governmental agencies – including the Swedish Public Health Agency (folkhalsomyndigheten.se), the National Board for Health and Welfare (socialstyrelsen.se), the Civil Contingencies Agency (msb.se), and the Work Environment Authority (av.se) – as well as newspaper and news media archives belonging to dailies Dagens Nyheter and Svenska Dagbladet, English-speaking Swedish online newspaper The Local, and public broadcasters Sveriges Radio (SR) and Sveriges Television (SVT) were consulted and materials related to the COVID-19 pandemic were collected and read through in order to identify materials of relevance. The selection criteria were that the material should describe an intervention, policy decision, public health recommendation, or event related to the government's management of the COVID-19 pandemic and/or its political, economic, social, or public health consequences. Materials detailing the same event where either combined or priority was given to the datum providing more detail on what took place, when it occurred, and who was involved.
Data source location	Lund University Lund Sweden Primary data sources: World Health Organization's webpages: who.int European Centre for Disease Prevention and Control´s webpages: ecdc.europe.eu The Swedish Government's webpages: regeringen.se The Swedish Riksdag´s webpages: riksdagen.se The Swedish Royal Court´s webpages: kungahuset.se The Swedish Public Health Agency's webpages: folkhalsomyndigheten.se The Swedish National Board of Health and Welfare's webpages: socialstyrelsen.se The Swedish Medical Products Agency's webpages: mpa.se The Swedish Pharmaceutical Industry Insurance Company's webpages: lff.se The Swedish Civil Contingencies Agency´s webpages: msb.se; krisinformation.se The Swedish Health and Social Care Inspectorate´s webpages: ivo.se
	The Swedish Agency for Health Technology Assessment and Assessment of Social Services’ webpages: sbu.se Statistics Sweden's webpages: scb.se The Swedish Armed Forces’ webpages: forsvarsmakten.se The Swedish Defense Research Agency's webpages: foi.se The Swedish Work Environment Authority's webpages: av.se The Swedish Employment Agency's webpages: arbetsformedlingen.se The Swedish Transport Agency's webpages: trafikverket.se The National Agency for Education´s webpages: skolverket.se The Swedish Central Bank, the Riksbank´s webpages: riksbank.se The Swedish Postal Service´s webpages: postnord.se Stockholm region health authorities’ webpages: sll.se Stockholm Administrative Court's webpages: domstol.se City of Malmö´s webpages: malmo.se Royal Swedish Academy of Sciences’ webpages: kva.se Swedish Association for Long-Covid patients’ webpages: covidforeningen.se Public broadcaster SR's online news archive: sr.se Public broadcaster SVT's online news archive: svt.se National news media organizations: Dagens Nyheter, Svenska Dagbladet, Göteborgsposten, Expressen, Aftonbladet, Dagens Industri and The Local's online editions: dn.se; svd.se; gp.se; expressen.se; aftonbladet.se; di.se; thelocal.se
	International news media: Forbes, Washington Post, Politico, and the BBC: forbes.com; washingtonpost.com; politico.com; bbc.com Links to all materials used are included in the database. Materials from other sources feature occasionally and links are included in the database.
Data accessibility	Repository name: Zenodo Data identification number: DOI: 10.5281/zenodo.5718433Direct URL to Data: https://doi.org/10.5281/zenodo.5718433

## Value of the Data

• This dataset offers a detailed catalogue of government policies, interventions, and events related to the COVID-19 epidemic in Sweden in the period from when the WHO first picked up on reports of an outbreak of pneumonia of unknown cause December 31, 2019, to the first steps to ease restrictions and recommendations in Sweden June 3, 2021. Database entries consist of summary descriptions of each intervention or event detailing what took place, when it happened, who was involved, and provides links to primary sources for each entry.
• The dataset will benefit researchers working on topics related to the COVID-19 pandemic, the COVID-19 epidemic in Sweden including topics related to policymaking, governance, government, and crisis management. The dataset will also benefit anyone on or interested in the Swedish strategy for managing COVID-19.
• Cataloguing interventions and events over time, the dataset provides a saturated and detailed account of government interventions and events in Sweden during the pandemic and offers both an overview of what took place in the country during the period described here and a resource for researchers working on Swedish governance during the pandemic as well as a strong basis for comparative work on Sweden and other jurisdictions.

## 1 Data Description

The dataset  contains details on the date an event took place (column 1), tags to facilitate navigation (column 2), details on the principal actors involved in the event (column 3), a summary description of what took place and who was involved (column 4), and links to primary materials (e.g., archival entries) (columns 4–12). Through a structured and detailed outline, the dataset provides a saturated account of policy interventions and events in Sweden during the 2020-21 global COVID-19 pandemic and complements existing and less detailed timelines published at earlier points in the period [1]. Moreover, by providing a detailed qualitative account of a single case, the dataset also complements quantitative and cross-national datasets such as the Oxford COVID-19 Government Response Tracker [2]. Finally, the dataset also complements the epidemiological data made available, e.g., by the European Centre for Disease Prevention and Control [3], and the Swedish Public Health Agency [4]. The dataset is meant to offer a catalogue of what took place in terms of governance in Sweden during the pandemic and to enable in-depth comparison of developments, policy interventions, and strategies between different jurisdictions.

The Swedish strategy's emphasis on voluntary measurements and citizen responsibility [5,6] made the country's approach to managing COVID-19 stand out. In a world where many countries opted for lockdowns to limit mortality and morbidity from COVID-19, Sweden chose a slightly different path to reach the same targets [7]; and saw harsh criticism for doing so [8]. Because of the attention and criticism leveled against the Swedish management of COVID-19, a detailed dataset on what measures the government and its agencies introduced during the pandemic is of essential necessity in future research as researchers will have to balance accounts of what took place against artefacts stemming from the substantial amount of negative media attention the Swedish strategy received both domestically [9] and abroad [10]. Good, detailed qualitative data will also be helpful in balancing the many premature assessments that scholars have made of Sweden's handling of the pandemic (for an extreme case see Lindström's commentary on the Swedish strategy [11,12]). We are confident that high quality qualitative data will be of importance for future research on how different countries and jurisdictions handled the pandemic and what effects it had on the many crises the pandemic gave rise to [13]. Our ambition is that this timeline will be of some help in doing so.

## 2 Experimental Design, Materials and Methods

The dataset was assembled by building up a catalogue of events as they unfolded over the course of the COVID-19 epidemic in Sweden (e.g., as the government or its agencies announced new measures or interventions) and by follow up reviews of government and media archives for the period December 31, 2019, to June 3, 2021. Online archives belonging to the Swedish Government (regeringen.se), the Swedish Public Health Agency (folkhalsomyndigheten.se), the National Board of Health and Welfare (socialstyrelsen.se), the Civil Contingencies Agency (msb.se), and the Work Environment Authority (arbetsmiljoverket.se) were investigated for entries related to the COVID-19 pandemic. Complementary reviews were also made of other agencies’ archives when those agencies featured as parts in a policy, intervention, or event. Newspaper archives for the largest Swedish daily newspapers Dagens Nyheter and Svenska Dagbladet, the Swedish English-language newspaper The Local, as well as archives belonging to public broadcasters Sveriges Radio (SR) and Sveriges Television (SVT) were also investigated for complementary materials. Materials were selected for inclusion based on assessment of whether the material described an intervention, policy decision, public health recommendation, or event related to the government's management of the COVID-19 pandemic and/or its political, economic, social, or public health consequences. In cases when two or more objects described the same event, objects were combined, and multiple sources were listed in the reference columns. When one datum offered significantly more details on what took place, when it occurred, and who was involved, the more detailed object was kept and the less detailed left aside.

While efforts have been made to ensure that the dataset provides a fair and accurate representation of events, policies, and interventions relevant to the COVID-19 epidemic in Sweden, the dataset contains some limitations. First, while we have been able to collect information on events, policies, and interventions, we have not been able to collect as much data on their long-term outcomes. This in part because the pandemic is still unfolding, and in part because of what information is available in the archives investigated when constructing this dataset. Second, because the dataset contains qualitative data, its construction has involved an inevitable element of subjective judgement. We have attempted to reduce the risk of bias by: (i) by joint reflection and critical discussion of how data is to be selected and recorded, and (ii) by using several sources to triangulate data entries whenever possible. Finally, we wish to highlight the possibility that some events may have failed to get registered in governmental or newspaper archives wherefore it is possible that they have also been overlooked in this dataset.

## CRediT authorship contribution statement

**Tobias Olofsson:** Data curation, Methodology, Writing – review & editing. **Andreas Vilhelmsson:** Conceptualization, Methodology, Data curation.

## Declaration of Competing Interest

This research was funded by a grant from the Faculty of Social Sciences at Lund University, Sweden. The authors declare that they have no known competing interests or personal relationships that could have appeared to influence the work reported in this article.

## Appendix Supplementary materials

Image, application 1
